# Mindfulness‐based cognitive therapy for chronic noncancer pain and prescription opioid use disorder: A qualitative pilot study of its feasibility and the perceived process of change

**DOI:** 10.1002/brb3.3005

**Published:** 2023-05-24

**Authors:** Hannah Ellerbroek, Imke Hanssen, Karen Lathouwers, Linda Cillessen, Sander Dekkers, Stijn E. Veldman, Sandra A. S. van den Heuvel, Anne E. M. Speckens, Arnt F. A. Schellekens

**Affiliations:** ^1^ Department of Psychiatry Radboud University Medical Center Nijmegen The Netherlands; ^2^ Nijmegen Institute for Scientist‐Practitioners in Addiction (NISPA) Nijmegen The Netherlands; ^3^ Department of Psychiatry, Centre for Mindfulness Radboud University Medical Centre Nijmegen The Netherlands; ^4^ Donders Institute for Brain, Cognition and Behaviour Radboud University Nijmegen The Netherlands; ^5^ Novadic‐Kentron Addiction Care Vught The Netherlands; ^6^ Department of Anesthesiology, Pain and Palliative Medicine Radboud University Medical Centre Nijmegen The Netherlands

**Keywords:** addiction, pain, psychiatric disorders, psychiatry, mindfulness

## Abstract

**Background:**

Mindfulness‐based interventions have a positive impact on pain, craving, and well‐being in both patients with chronic pain and those with opioid use disorder (OUD). Although data are limited, mindfulness‐based cognitive therapy (MBCT) might be a promising treatment for patients with chronic noncancer pain combined with OUD. The aim of this qualitative study was to explore the feasibility and process of change during MBCT in this particular population.

**Methods:**

In this qualitative pilot study, 21 patients who were hospitalized for rotation to buprenorphine/naloxone as agonist treatment for chronic pain and OUD were offered MBCT. Semistructured interviews were conducted to explore experienced barriers and facilitators to MBCT. Patients who participated in MBCT were also interviewed on their perceived process of change.

**Results:**

Of 21 patients invited to participate in MBCT, 12 initially expressed interest but only four eventually participated in MBCT. The timing of the intervention, group format, somatic complaints, and practical difficulties were identified as the main barriers to participation. Facilitating factors included having a positive attribution toward MBCT, an intrinsic motivation to change, and practical support. The four MBCT participants mentioned several important mechanisms of change, including reduction of opioid craving and improved coping with pain.

**Conclusions:**

MBCT offered in the current study was not feasible for the majority of patients with pain and OUD. Changing the timing of MBCT by providing it at an earlier stage of the treatment and offering MBCT in an online format may facilitate participation.

## Graphical abstract

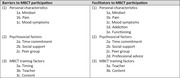

## 1 INTRODUCTION

Although opioids are potent analgesics, there is no evidence supporting long‐term use in chronic noncancer pain (Nury et al., 2022). Yet, a substantial proportion of patients with chronic pain are treated with opioids—in the United States, around 15% (Groenewald et al., 2022). It has been estimated that around 10% of patients who use prescription opioids for chronic pain develop an opioid use disorder (OUD) (Vowles et al., 2015), characterized by an urge to use opioids (craving), failure to cut down on opioid use, and impairment in daily activities (Els et al., 2017). Next to their analgesic effects, some patients also report the use of opioids to cope with negative emotions (Wasan et al., 2007). Indeed, anxiety and depression frequently co‐occur in patients with chronic pain (van Rijswijk et al., 2019), and opioid use itself also increases the risk for developing mood symptoms (Leung et al., 2022). Together, this may accumulate into a downward spiral of pain, negative emotions, craving, and increasing prescription opioid use (Garland, 2014).

In order to break this downward spiral, psychological treatment aimed at coping with pain, craving, and negative emotions might be helpful to improve quality of life and reduce opioid use. Mindfulness‐based interventions (MBIs) may be specifically suitable for this population by helping patients to allow negative emotions, including pain and mood symptoms, and replace automatic reactions, including opioid use, by more conscious and helpful responses. MBIs can cultivate specific mindfulness skills, such as self‐awareness, acceptance, and self‐compassion (Garland, 2014; Garland et al., 2012; Kim et al., 2018). During MBIs, patients practice to be aware in the present moment and to have a nonjudgmental attitude to their current experience (Kabat‐Zinn & Hanh, 2009).

MBIs have repeatedly been shown effective in improving coping with negative emotions, both in patients with mood and anxiety disorders (Goldberg et al., 2018) and in patients with chronic pain (Chiesa & Serretti, 2011; Esmer et al., 2010; Garland et al., 2014, 2012). Studies specifically focusing on patients who use prescription opioids for pain are promising; a small study reports pain reduction with an effect size of 0.86 (Zgierska, Burzinski, Cox, Kloke, Stegner, et al., 2016). Other studies find moderate effect sizes on reducing craving and opioid use (Cooperman et al., 2021; Garland et al., 2014, 2016).

However, some patients experience barriers toward participation in an MBI, including barriers related to pain and organizing transportation (Zgierska, Burzinski, Cox, Kloke, Singles, et al., 2016). Furthermore, most studies focus primarily on patients with chronic pain but without OUD. It is currently unknown whether these MBIs are equally appropriate for the treatment of patients with both chronic pain and OUD. Given the limited data on MBIs for patients with chronic pain combined with prescription OUD and its potential effectiveness, the aim of this pilot study was to assess the feasibility of mindfulness‐based cognitive therapy (MBCT) in this population. It is essential to assess the ability and willingness of patients to participate in this treatment and identify any adaptations that need to be made before conducting larger studies. Our secondary aim was to assess the process of change for patients who participated in the MBI to get an initial indication of its effectiveness. Our study was conducted in a patient group that was offered agonist treatment with buprenorphine/naloxone (BuNa) prior to the MBI.

## 2 METHODS

### 2.1 Study design

A qualitative pilot study was conducted to assess feasibility and process of change for MBCT in patients with chronic pain and OUD. The study was performed at the Department of Psychiatry at Radboud University Medical Center, Nijmegen, the Netherlands. Ethical approval was waived by the regional ethical committee (CMO Arnhem‐Nijmegen) based on the Dutch Medical Research Involving Human Subject Act (WMO), as MBCT is already part of regular clinical care at our department. According to the WMO, studies on regular clinical care are exempted from ethical approval, as long as burden for participation is minimal. The regional ethical committee assessed the study protocol (protocol no. 2015‐1551) and concluded that it met these criteria for exemption.

All patients scheduled for BuNa rotation were informed on the possibility to participate in the study during hospitalization (see flowchart Appendix S1), either face‐to‐face or over phone. Patients who agreed to participate in the study gave written informed consent prior to data collection and were explained that MBCT would be scheduled 3 months after the rotation. Patients did not receive financial compensation for their participation.

### 2.2 Participants

Participants were recruited among patients referred for opioid agonist treatment with BuNa. Participants were adults, met ICD‐11 criteria for chronic pain (persistent or recurring noncancer pain lasting ≥3 months) (Treede et al., 2019), and met the Diagnostic and Statistical Manual of Mental Disorders (5th edition) criteria for OUD (American Psychiatric Association, 2013). Patients were excluded if they met somatic contraindications for BuNa treatment, if they had severe cognitive impairments, or if they had acute psychiatric comorbidity such as psychosis or acute suicidality.

### 2.3 Measurements

Prescription OUD as well as concurrent substance use was assessed using the Measurements in the Addictions for Triage and Evaluation (MATE). The MATE is a clinician‐administered interview used in Dutch addiction care (Schippers et al., 2010). Presence of other psychiatric conditions according to DSM‐IV was assessed using the Mini International Neuropsychiatric Interview Plus (MINI‐plus) (van Vliet & de Beurs, 2007).

### 2.4 Intervention

MBCT was based on the original MBCT protocol for recurrent depression of Segal et al. (2018). It consisted of eight weekly sessions of 2.5 h, plus a 6‐h practice silent day. Sessions included formal meditation exercises, such as a body scan meditation, sitting meditation, walking meditation, and mindful movement, and informal meditation exercises, such as doing daily activities with awareness. In addition, the program included cognitive techniques such as psychoeducation, monitoring, scheduling activities, and identification of negative automatic thoughts. Next to that, participants were instructed to practice mindfulness for 45 min a day at home with the help of guided exercises (audio files) and to complete other homework assignments (such as mindful awareness to everyday activities). MBCT was taught by one experienced mindfulness teacher and one teacher in training (K.L.), meeting the advanced criteria of the Association of Mindfulness‐Based Teachers in the Netherlands and Flanders and the internationally agreed good practice guidelines of the UK Network for Mindfulness‐Based Teachers (Crane et al., 2012). Group size varied between eight and 10 participants. MBCT groups were open to all patients with psychiatric conditions and not specifically focused on addiction.

### 2.5 Procedure

Prior to BuNa rotation, baseline characteristics were assessed, including the MINI‐plus and MATE. Following BuNa rotation, a semi‐structured interview on the perceived barriers and facilitators to participate in MBCT was conducted. In patients who did not participate in an interview, reasons for (not) participating in MBCT were still collected. Hereafter, patients who decided to participate in MBCT started the 8‐week MBCT. Subsequently, the MBCT participants were invited for a second semistructured interview on their perceived process of change during MBCT.

### 2.6 Qualitative interviews

Patients were interviewed in a semistructured manner. In the first interview, prior to start of MBCT, patients were asked (1) whether they wanted to participate in MBCT, (2) their motives for (not) participating, and (3) possible barriers and facilitators to participation. Questions were followed by probes to gain more insights about the barriers and facilitators, and were asked in an open nondirective manner, allowing patients to speak freely. Some patients who first agreed to participate in MBCT and later changed their mind were interviewed twice (see Appendix S2). Most interviews took between 30 and 60 min and were conducted in the clinic. Some interviews were conducted by video call or phone due to the COVID‐19 pandemic. Only the interviewee and interviewer were present. No notes were made. Interviews were audiotaped and transcribed verbatim. The first 10 interview transcripts were returned to the participants. Patients who participated in MBCT were invited for a second interview about their perceived process of change. Interviews were conducted by K.L., L.C. (both psychologists/researchers), or A.E.M.S. (psychiatrist), one‐on‐one with the participant. All interviewers were female. L.C. and A.E.M.S. are both experienced mindfulness teachers, and K.L. was finalizing her training to be a mindfulness teacher. Patients knew that the interviewers were studying feasibility and effectiveness of the intervention. They did not provide feedback on the interviews or the findings. Reporting of the qualitative study included the elements of the Consolidated Criteria for Reporting Qualitative Studies (COREQ) checklist (Tong et al., 2007).

### 2.7 Data analysis

Data analysis was done using a thematic analysis approach (Braun & Clarke, 2006). Two researchers joined the study for data analysis: another psychologist/researcher/qualified mindfulness teacher (I.H.) and a PhD candidate focusing specifically on patients with chronic pain and prescription OUD (H.E.). Transcripts were coded independently in pairs of two researchers (K.L., L.C., I.H., and H.E., in different combinations) using ATLAS.ti (Version 22.0.11) (ATLAS.ti Scientific Software Development GmbH, 2022). Codes were compared and discussed between two researchers until agreement was reached. After each coding session, the list of codes was adapted for the next coding session. Codes were grouped into themes during two team meetings, the first of which took place after 13 of 22 interviews were conducted. The first thematization meeting was attended by K.L. and A.E.M.S., as well as a psychiatrist (A.F.A.S.) and a physician assistant (S.D.) specialized in addiction treatment. The second thematization meeting was done after data collection and coding were finished. The same four team members and three additional team members joined this meeting; I.H., H.E., and anesthesiologist specialized in patients with chronic pain (S.H.). After thematization and data analysis, we selected illustrative quotes and translated them into English. Data saturation was reached regarding the interviews on feasibility, but not regarding the interviews on effectivity of MBCT.

## 3 RESULTS

### 3.1 Study flow

We screened 39 patients from clinical admission for BuNa rotation between April 2020 and July 2021, of which 24 patients successfully participated in BuNa rotation and 21 patients agreed to be included in data collection for this study. Nine (43%) out of the 21 patients did not want to participate in MBCT. The primary reasons for this were having already participated in an MBI before, having too much pain, too much travel distance and time, and not being interested in psychosocial interventions. Three patients out of this group were interviewed to further explore their barriers to participation. The study flow is also described in Appendix S1.

Twelve patients initially expressed interest in MBCT, but eight of these patients refrained from participating in the 3 months before the start of MBCT. From these eight patients, two patients were only interviewed during their first moment of choice (when being positive about participation), two were interviewed only during their second moment of choice (when refraining from participation), and four were interviewed at both these timepoints. In Appendix S2, it is described which patients participated in how many interviews. The primary reasons for choosing not to participate later in the process were MBCT being too late within the treatment process and practical difficulties.

Four patients (19%) participated in MBCT. Three out of these patients were interviewed prior to MBCT about barriers and facilitators. One patient participated in all eight sessions, two patients participated in seven sessions, and one patient participated in six sessions. All four patients attended the silent day. All four patients were interviewed to explore their perceived process of change.

### 3.2 Baseline characteristics of interviewed population

Fifteen of 21 patients participated in at least one qualitative interview. The baseline demographic and clinical characteristics of the interviewed population (*n* = 15) can be seen below in Table 1 (see Appendix S3 for a description of the entire population).

**TABLE 1:** Baseline demographic and clinical characteristics.

Patient characteristics	Total (*n* = 15)
Demographics	
Gender (F/M)	9/6
Age (mean, *SD*)	47.8 (12.1)
Cohabiting	10 (67%)
Employed	2 (13%)
Educational level^a^	
Low	5 (33%)
Intermediate	7 (47%)
High	3 (20%)
Pain at baseline	
Type of pain	
Neuropathic	13 (86%)
Idiopathic	1 (7%)
Nociceptive	1 (7%)
Pain intensity, VAS‐score (mean, *SD*)	5.9 (1.9)
Mood symptoms	
Current depression	1 (7%)
Anxiety disorder	3 (20%)
Use of other medication	
Sedatives	5 (33%)
Use of other substances^b^	
Alcohol	4 (27%)
Nicotine	10 (67%)

Abbreviations: F, female; M, male; VAS, Visual Analog Scale (0–10).

^a^ Educational level is classified as low (no education, elementary school, or lower secondary education), middle (intermediate vocational education, upper secondary education), and high (higher vocational education, university).

^b^ Use of substance at least once a month.

Prior to BuNa rotation, patients used oxycodone (nine patients) or fentanyl (six patients), with an average oral morphine equivalent dose of 169 mg.

### 3.3 Barriers and facilitators of MBCT participation

From analysis of the interviews, three categories of barriers and facilitators emerged: (1) personal characteristics, (2) psychosocial factors, and (3) MBCT training factors (see Table 2). See Appendix S4 for illustrative quotes.

**TABLE 2:** Themes and subthemes of facilitators and barriers prior to mindfulness‐based cognitive therapy (MBCT) participation

Barriers to MBCT participation	Facilitators to MBCT participation
(1) Personal characteristics 1a. Mindset 1b. Pain 1c. Mood symptoms (2) Psychosocial factors 2a. Time commitment 2b. Social support 2c. Peer group (3) MBCT training factors 3a. Timing 3b. Teacher 3c. Content	(1) Personal characteristics 1a. Mindset 1b. Pain 1c. Mood symptoms 1d. Addiction 1e. Functioning (2) Psychosocial factors 2a. Time commitment 2b. Social support 2c. Peer group 2d. Professional advice (3) MBCT training factors 3a. Teacher 3b. Content

#### 3.3.1 Barriers

##### 3.3.1.1 Personal characteristics

###### Mindset

Barriers to MBCT participation were a negative view and low expectations of MBCT, an avoiding coping style regarding difficult feelings, and a somatic attribution of pain.

###### Pain

Some patients expected that pain or fatigue itself would hinder participation. They worried that somatic complaints would get worse during MBCT sessions, for example, by sitting in uncomfortable poses for longer periods. A long travel distance also served as a barrier, as sitting in a car worsened pain. One of the MBCT participants mentioned that some days he had too much pain to meditate.

###### Mood symptoms

Having depressive feelings and anxiety hindered participation. Some patients were worried that their mood symptoms would get worse during or after MBCT.

##### 3.3.1.2 Psychosocial factors

###### Time commitment

Some patients, especially if they had other responsibilities such as work or childcare, considered the number and duration of the meetings, the homework, or the silent day as being too much. One of the four MBCT participants found it difficult to practice meditation during busy periods.

###### Social support

Lack of social support, such as feeling alone or having no help with transportation to the training, also hindered participation.

###### Peer group

Some patients anticipated that there would be a lack of privacy in the peer group, were worried about contracting a COVID‐19 infection, or had social anxiety and experienced shame doing exercises or sharing their experience in a group. There were also two patients indicating they were easily irritated by other people.

##### 3.3.1.3 MBCT training factors

###### Timing

Multiple patients considered MBCT as being too late within their treatment process and preferred to receive MBCT during their hospitalization. First, patients expected to have more free time to practice with mindfulness during hospitalization than at home. Second, patients indicated that it would have been helpful to receive MBCT before or during hospitalization, as mindfulness skills, such as refocusing attention, would have been helpful during the most severe moments of withdrawal and craving during opioid rotation.

###### Teacher

One patient only wanted to participate if the teacher had life experience.

###### Content

A frequently mentioned barrier was already having experienced an MBI before. One of the patients who had chosen to participate in MBCT had difficulties understanding the reader.

#### 3.3.2 Facilitators

##### 3.3.2.1 Personal characteristics

###### Mindset

A positive view and expectations of MBCT, a holistic approach toward pain, having an open mind, and taking on every opportunity to improve the current situation were facilitators to participate in MBCT. Being solution driven, goal oriented, and flexible and getting easily along with other people also facilitated participation.

###### Pain

Patients hoped that MBCT would reduce their pain, teach them to accept and cope with pain better, and provide distraction from the pain.

###### Mood symptoms

Almost all patients talked about learning how to cope with anxiety and worries, and to accept themselves more. Two patients expressed the wish to be more present and enjoy the current moment. Most patients wanted to learn how to protect their boundaries. They hoped that MBCT would reduce overreacting to emotions and make them less chaotic and irritable. Wishing to reduce stress was mentioned as well.

###### Addiction

Patients hoped MBCT would reduce or distract from craving for and dependency to their medication. They also wanted to learn how to deal with withdrawal symptoms. One patient wanted to communicate more openly about opioid use and OUD. One patient participated in MBCT to prevent relapse into opioid use.

###### Functioning

Participants mentioned that they wanted to get their old life or old “sense of me” back and to improve communication with others. They hoped to live a more valuable life, for example, by starting to work again. Patients furthermore wanted to increase their attention span and organize their thoughts.

##### 3.3.2.2 Psychosocial factors

###### Time commitment

For some patients, the duration of the meetings fitted well into their day. One patient mentioned that since she was no longer employed, she had enough time to practice MBCT.

###### Social support

Being supported with practicalities, for example, help with driving and childcare duties, facilitated participation. Motivational support also facilitated participation, such as friends having a positive attitude toward MBCT.

###### Peer group

The peer group motivated some patients. They expected to feel recognition, to understand each other, and to be around motivated people. The four patients who eventually participated in MBCT all said that the peer group provided company and recognition, and that they learned from each other during the training.

###### Professional advice

MBCT being presented as part of the treatment protocol and being advised to participate in MBCT by a professional both facilitated participation.

##### 3.3.2.3 MBCT training factors

###### Teacher

The four MBCT participants valued the open‐minded and accepting attitude of the teacher.

###### Content

All four patients experienced practicing mindfulness at home helpful, with three out of the four patients continuing meditation practice after the MBCT training ended. Patients mentioned the partner session, the silence day, the at‐home audio files, the laying down movement exercises, and the body scan meditation as useful MBCT elements. During MBCT, they liked that they were able to adapt exercises to their own needs. The time required for the training was also acceptable.

#### 3.3.3 Process of change during MBCT

For the four MBCT participants, we identified a number of changes (see Table 3). See Appendix S4 for illustrative quotes. For this patient group, data saturation could not be reached due to the small number of participants in MBCT.

**TABLE 3:** Process of change during mindfulness‐based cognitive therapy (MBCT)

Process of change following MBCT
(1) General changes 1a. Emotion regulation 1b. Thoughts 1c. Behavior1d. Interpersonal (2) Specific to population 2a. Pain 2b. Addiction

##### 3.3.3.1 General changes

###### Emotion regulation

Patients expressed that they were more in touch with and observant of their emotions after MBCT. One patient's anxious emotions had diminished. Another patient who struggled with anger mentioned that this had reduced as well. Patients reported enjoying more moments of calmness and happiness.

###### Thoughts

Patients expressed that they became better in accepting the current situation as it is. All patients felt calmer than before MBCT, and one patient slept better. Furthermore, patients were more compassionate toward themselves, such as putting less blame on themselves than before. All patients reported that they could distance themselves from their thoughts. Two patients mentioned that they felt less “mental chaos.” One patient experienced an improved ability to focus attention.

###### Behavior

Patients expressed they learned to set boundaries, plan better, and take rest when needed. Another patient became better in dealing with feelings of restlessness. Patients also reported participating more in activities that they enjoyed.

###### Interpersonal

Patients described that after MBCT, it was easier to talk with others about their problems, to be more honest and open about how they were feeling. One patient experienced more empathy toward others and one participant expressed feeling like “herself” again after MBCT (happier and calmer), which was confirmed by her partner.

##### 3.3.3.2 Specific to population

###### Pain

Two patients mentioned that participation in MBCT had no direct effect on their pain. One patient, however, said that MBCT helped in creating a feeling of calmness, and that this feeling reduced pain levels. He had realized that having stress also led to headaches. All patients expressed that they became better in dealing with pain, either by letting it go, by looking for distractions, or by accepting it. Patients also became aware of the influence of stress on pain. One patient expressed that the body scan meditation was helpful in focusing on where in the body she felt pain and accepting it. Another patient experienced his daily life was less affected by pain than prior to MBCT, and that he was part of society again.

###### Addiction

A patient expressed that when being stressed, he normally would crave for opioids. MBCT helped to reduce stress and in this way reduced craving. This patient also expressed an increased ability to cope with craving by reflecting on the craving, realizing why he was craving for opioids, and being aware of the negative consequences of giving in to the craving. Patients also said MBCT helped to focus on something else when experiencing craving or opioid withdrawal symptoms, such as a body scan meditation.

## 4 DISCUSSION

This pilot study investigated the feasibility and perceived process of change of MBCT as an add‐on treatment after agonist treatment with BuNa in patients with chronic pain and prescription OUD. An important finding is that most patients (17 out of 21) chose not to participate in MBCT. Although MBCT can have beneficial effects, offering MBCT in its current format does not seem particularly feasible for this patient population. By understanding the barriers to participation and reducing these barriers, more patients may choose to participate in MBCT in the future.

### 4.1 Barriers, facilitators, and implications

We offered MBCT 3 months after initiating BuNa treatment. A frequently mentioned barrier to participation was that MBCT was offered late within the treatment process. Offering MBCT earlier might mean that patients could use mindfulness skills to deal with difficult moments of increased pain or craving during pharmacotherapy. Indeed, in a recent pilot study, 15 patients participated in Mindfulness‐Oriented Recovery Enhancement (MORE) during methadone substitution therapy. Unlike our population, these patients were primarily using illicit opioids at baseline, but they also all had chronic pain. The MORE group was compared with a group being offered counseling. In both treatment arms, there was little dropout during this period (Cooperman et al., 2021), indicating that this timing is feasible for an MBI. Furthermore, patients participating in MORE, as addition to rotation to methadone, had more reductions in craving, illicit opioid use, and pain compared to patients rotating to methadone with counseling (Cooperman et al., 2021). In future research on MBCT in patients with chronic pain and OUD, MBCT could be offered in adjunct to rotation to opioid agonist therapy or any other intervention, such as tapering. As patients in our study mentioned, they may use coping skills that they learn during the MBI to cope with pain or craving during the change in medication.

Other barriers included mental complaints and the fear that pain and mental complaints would get worse with MBCT, and not wanting to participate in a group. Regarding these fears, giving patients the opportunity to try out a single MBCT session may be a solution to take some worries away.

There were also practical barriers to participation: having other responsibilities, limited social support, too much pain, and too many mental complaints. Having limited time and practical/social support have also been identified as barriers in other studies on feasibility of MBIs in patients using opioids for chronic pain (Zgierska, Burzinski, Cox, Kloke, Singles, et al., 2016) as well as in different populations with somatic or psychiatric complaints (Brintz et al., 2020; Ewais et al., 2020; Hanssen et al., 2020; Janssen et al., 2020; Schoultz et al., 2016). To overcome these practical issues, MBCT could be offered in an individual, online format, where patients can follow the eight sessions in their own pace from home. A cross‐sectional survey with 500 respondents in the United States showed that 80% of participants preferred an individual and/or online format of MBIs. Most of the respondents to the survey had depression and/or posttraumatic stress disorder. Electronic MBCT could remove barriers by allowing patients to participate in their own environment, in their own pace, and without travelling. Furthermore, there are generally no waiting lists for online interventions and they are often inexpensive (Spijkerman et al., 2016). For patients with (chronic) pain, anxiety, and depression, the effect sizes for stress reduction are similar for online MBIs compared to traditional in‐person group formats (Spijkerman et al., 2016). Limitations of online interventions are relatively high dropout rates and more difficulty engaging participants compared to an in‐person intervention (Mrazek et al., 2019). Yet, for patients who otherwise do not participate in any training due to somatic complaints, anxiety, or practicalities, online MBCT can be a solution.

Ultimately, next to patients who experienced these difficulties, there was also a subset of patients in our study who were simply not interested in MBCT. For some patients, psychoeducation aimed at understanding their pain better may be a first step toward an MBI. For other patients, a psychological intervention may just not be the most fitting treatment.

### 4.2 Perceived process of change

While our study gives insight in the barriers and facilitators to participation, we are limited in drawing conclusions about the effectiveness of the intervention. The four MBCT participants indicated that changes in their pain coping, opioid craving, and an increased sense of functioning contributed to their recovery process. They all perceived MBCT as useful. Improved pain coping has also been reported in another qualitative study in 21 patients with chronic low back pain and long‐term opioid use participating in an MBI (Zgierska, Burzinski, Cox, Kloke, Singles, et al., 2016). Yet, quantitative studies with more patients are necessary to draw conclusions about the effectiveness of MBCT in this patient population.

### 4.3 Strengths and limitations

A strength of our study is the representative sample of patients from clinical practice that we asked to participate in our study, allowing us to investigate the barriers to participation in depth. A limitation, however, is that due to the limited number of MBCT participants, data saturation was not achieved regarding our questions on the process of change during MBCT.

## 5 CONCLUSION

Although the participants who completed the MBCT were generally positive, MBCT in the current form is not feasible for the majority of the patient population. We identified multiple barriers to MBCT participation, including timing of the intervention and practical difficulties. Changing the timing of the MBCT by providing it at an earlier stage of the treatment and offering MBCT in an online format may facilitate participation. Furthermore, psychoeducation and giving patients the opportunity to try out a single MBCT session might support them to make a decision toward participation too.

## AUTHOR CONTRIBUTIONS

Karen Lathouwers, Anne E. M. Speckens, and Arnt F. A. Schellekens designed the study. Anne E. M. Speckens was the principal investigator for this study regarding the MBCT. Arnt F. A. Schellekens was the responsible physician and principal investigator for BuNa rotation. Karen Lathouwers, Linda Cillessen, and Anne E. M. Speckens conducted the qualitative interviews. Karen Lathouwers and Sander Dekkers collected quantitative data. Karen Lathouwers, Linda Cillessen, Imke Hanssen, Hannah Ellerbroek, Sandra A. S. van den Heuvel, Sander Dekkers, Anne E. M. Speckens and Arnt F. A. Schellekens contributed to data analysis. Hannah Ellerbroek, Imke Hanssen, and Karen Lathouwers developed the initial draft of the manuscript after which all authors read, revised, and supplemented the draft of the manuscript and agreed to the final version.

## CONFLICT OF INTEREST STATEMENT

The authors declare no conflicts of interest.

### PEER REVIEW

The peer review history for this article is available at https://publons.com/publon/10.1002/brb3.3005.

## Supporting information

Supp Information

Supp Information 2

## Data Availability

The data that support the findings of this study are available from the corresponding author upon reasonable request.
