# Supplementary material for: Mindfulness‐based cognitive therapy for chronic noncancer pain and prescription opioid use disorder: A qualitative pilot study of its feasibility and the perceived process of change
Source: Brain Behav. 2023 May 24;13(7):e3005. doi: 10.1002/brb3.3005 (PMC10338789; doi:10.1002/brb3.3005)
Supplement: Supplementary file 1 — Supp Information [file BRB3-13-e3005-s001.docx]

**
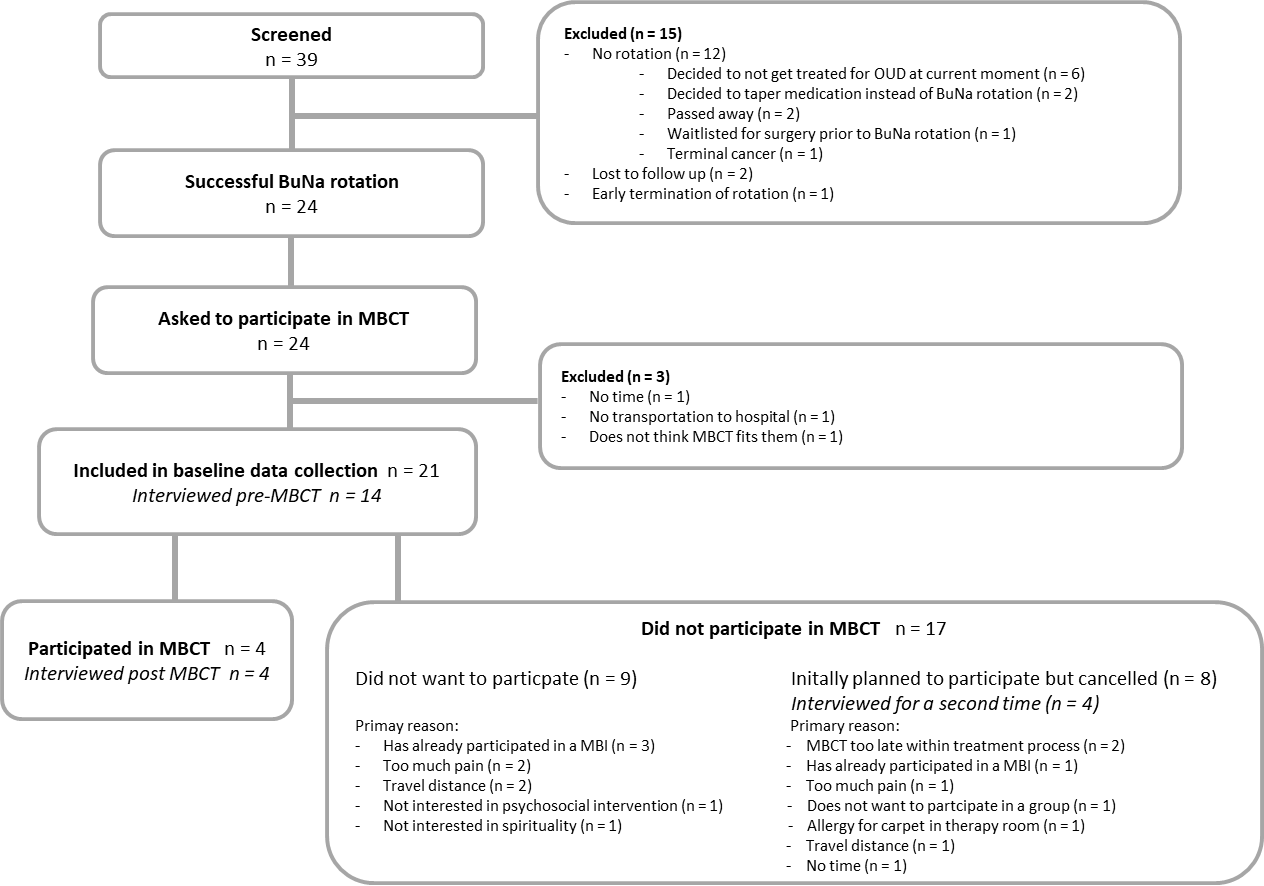
Appendix 1: Study flow chart**

Figure S1: Study flow chart

**Appendix 2: Interviewed patients**

| **Patient** | **Gender** | **Age** | **Interviewed prior to MBCT** | **MBCT participation** | **Interviewed after MBCT** |
| --- | --- | --- | --- | --- | --- |
| P1 | Male | 62 | 1x | No | - |
| P2 | Male | 23 | - | Yes | 1x |
| P3 | Male | 40 | 1x | No | - |
| P4 | Female | 58 | 1x | No | - |
| P5 | Female | 48 | 2x | No | - |
| P6 | Female | 45 | 2x | No | - |
| P7 | Female | 64 | 1x | Yes | 1x |
| P8 | Male | 62 | 1x | No | - |
| P9 | Male | 41 | 1x | Yes | 1x |
| P10 | Female | 51 | 1x | No | - |
| P11 | Female | 52 | 2x | No | - |
| P12 | Female | 28 | 1x | No | - |
| P13 | Male | 53 | 2x | No | - |
| P14 | Female | 40 | 1x | Yes | 1x |
| P15 | Female | 47 | 1x | No | - |

Table S1: Demographic information of interviewed participants, number of interview participated in, and MBCT participation.

**Appendix 3: patient characteristics of all study participants**

Patients’ characteristics at baseline of all study patients (n = 21) including patients that did participate in interviews (n = 15) and those who did not participate in interviews (n = 6), stratified by choice.

| **Patient charasteristics** | **Did not want to participate (n = 9)** | **Initially planned to participate, then refrained (n = 8)** | **MBCT**  **participants**  **(n = 4)** | **Total**  **(n = 21)** |
| --- | --- | --- | --- | --- |
| **Demographics** |  |  |  |  |
| Female gender | 4 (44%) | 6 (75%) | 2 (50%) | 12 (57%) |
| Age (mean, SD) | 58.8 (8.6) | 48.6 (10.0) | 42.0 (16.8) | 51.7 (12.3) |
| Cohabiting | 5 (56%) | 6 (75%) | 3 (75%) | 14 (67%) |
| Employed | 2 (22%) | 1 (13%) | 1 (25%) | 4 (19%) |
| Educational level ^a^ |  |  |  |  |
| *Low* | 4 (45%) | 2 (25%) | 2 (50%) | 8 (38%) |
| *Intermediate* | 2 (22%) | 5 (63%) | 1 (25%) | 8 (38%) |
| *High* | 3 (33%) | 1 (13%) | 1 (25%) | 5 (24%) |
| **Pain at baseline** |  |  |  |  |
| Pain intensity, VAS-score (mean, SD) | 65.8 (24.3) | 63.1 (13.9) | 55.3 (20.9) | 62.6 (19.6) |
| Pain coping, PCI-score (mean, SD) | 39.5 (10.2) | 39.7 (8.3) | 31.3 (13.2) | 37.9 (9.8) |
| **Opioid use at baseline** |  |  |  |  |
| Oral morphine equivalent dose at baseline in mg (mean, SD) | 190.0 (86.0) | 157.5 (97.2) | 140.0 (67.8) | 167.0 (85.7) |
| Opioid craving, VAS-score (mean, SD) | 33.4 (31.4) | 29.4 (27.6) | 21.5 (24.0) | 29.4 (27.4) |
| **Mood symptoms** |  |  |  |  |
| Current depression | 3 (33%) | 0 (0%) | 0 (0%) | 3 (14%) |
| Anxiety disorder | 5 (56%) | 2 (25%) | 0 (0%) | 7 (33%) |
| Suicideal ideation | 4 (44%) | 2 (25%) | 0 (0%) | 6 (29%) |
| Depression, anxiety and stress, DASS-21-score (mean, SD) | 50.8 (29.1) | 41.1 (27.1) | 22.0 (14.0) | 41.2 (27.0) |
| Quality of life, MHC-SF-score (mean, SD) | 32.6 (9.7) | 27.3 (14.0) | 40.8 (10.4) | 32.4 (12.1) |
| **Use of other medication** |  |  |  |  |
| Sedatives | 5 (56%) | 3 (38%) | 0 (0%) | 8 (38%) |
| **Use of other substances ^b^** |  |  |  |  |
| Alcohol | 2 (22%) | 2 (25%) | 1 (25%) | 5 (24%) |
| Nicotine | 4 (45%) | 5 (63%) | 3 (75%) | 12 (57%) |
| **Mindfulness skills** |  |  |  |  |
| Mindfulness skills, FFMQ-score (mean, SD) | 70.0 (14.0) | 67.6 (10.1) | 59.0 (12.7) | 66.8 (12.5) |
| Self-compassion, SCS-score (mean, SD) | 44.8 (7.0) | 49.3 (5.8) | 48.0 (7.9) | 47.1 (6.7) |

Table S2: Baseline demographic and clinical characteristics.
^a^ Educational level is classified as low (no education, elementary school or lower secondary education), middle (intermediate vocational education, upper secondary education), and high (higher vocational education, university).
^b^ Use of substance at least once a month.

The following questionnaires were conducted at baseline and are described in the table above; a VAS-scale was used to measure pain intensity and the Pain Coping Inventory (PCI) was used to asses pain coping (Kraaimaat & Evers, 2003); a VAS-scale was used to measure craving; depression, anxiety, and stress symptoms were measured with the Depression Anxiety Stress Scale (DASS-21) (de Beurs, Van Dyck, Marquenie, Lange, & Blonk, 2001); Quality of Life was measured with the Mental Health Continuum – short form (MHC-SF) (Lamers, Westerhof, Bohlmeijer, ten Klooster, & Keyes, 2011); Mindfulness-skills were assessed with the Five Facet Mindfulness Questionnaire – Short Form (FFMQ-SF). The FFMQ-SF measures the five facets of mindfulness; 1) observing, 2) describing, 3) acting with awareness, 4) non-judging and (5) nonreactivity (Bohlmeijer, ten Klooster, Fledderus, Veehof, & Baer, 2011); Self-compassion was measured with the Self-Compassion Scale – Short Form (SCS-SF) (Raes, Pommier, Neff, & Van Gucht, 2011).

**Appendix 4: Illustrative quotes from the interviews**

|  | **Barrier** | **Facilitator** |
| --- | --- | --- |
| **Personal characteristics** | | |
| **Mindset** | *“I wouldn't know how to change that [pain] with a different mindset. Because the pain dominates everything.” (P8).* | *"So everything that is available, I just try. (...) Look, I think that at least the pain also partly is caused by emotion and stress and such." (P10)*. |
| **Pain** | *“So if I'm going to sit or stand a little longer than I'm doing now, then it's going to take a lot of energy, which is going to increase the pain. And the longer I participate in something, the more the pain will intensify with the result that the day after or even two days after I still have a lot of pain.” (P3).* | *“Well I think it [mindfulness] may help against pain by being occupied with your head and less with your body, so as a bit of distraction”. (P15).* |
| **Addiction** | *-* | *"I hope it is going to work (...) These thoughts in my head always say "Oxycodone, Oxycodone", you know. (…) I am afraid that one day it will go wrong." (P9).* |
| **Mood symptoms** | *"And then [during MBCT] I'm going to have even more problems, (…) get even more depressed and even more overworked." (P3).* | *"I kept thinking all the time about what happened in my past…. Things like that I can't let go easily and it just stays in my head and I hope it [mindfulness] will help." (P9)*. |
| **Functioning** | *-* | *"I would just like to work again. And I hope this mindfulness will help me with that." (P13).* |
| **Psychosocial factors** | | |
| **Time commitment** | *"Maybe if the circumstances had been different, if my daughter had been a bit older and I wouldn't have had all those things like the dog now, I would have done it online." (P6).* | *"You receive homework everywhere. (...) I have nothing better to do I always say, I'm at home anyway.” (P14).* |
| **Social support** | *“Since my husband has passed away, I have to do everything by myself. And that's really hard. (…) Look if he would come with me then he would just say [first name] I will drive you" (P4).* | *"We are very flexible. My partner (…) he has to arrange things. (...) And otherwise grandpa and grandma are there who can jump in." (P14).* |
| **Peer group** | *"I'm also ashamed of the physical stuff. Because when you have to lie on one of those mats during mindfulness and sit cross-legged, I can’t do that with my hip. Then I worry how I will sit and that everyone will look at me." (P12).* | *"Well what I've been told is that those people [peer group] are basically the same as me. So that's a plus of course." (P13).* |
| **Professional advice** | *-* | *“I imagine that the psychiatrist offers it [MBCT] for a reason. Therefore, it can only support you in your recovery." (P5).* |
| **MBCT training factors** | | |
| **Timing** | *"You only start the mindfulness 3 or 4 months later. Then you have already been through the hardest part. And I think I would have liked it better when you do have difficult moments (...) That you take your mind off things a bit at that moment." (P6).* | - |
| **Teacher** | *“I do think it’s really important that there's not a rookie in front of me. (…) I think life experience is very important to convey something." (P15).* | *"She delivered it well. Was open to everything. (...). I clicked with her and with everybody." (P7).* |
| **Content** | *"Sometimes I read something [in the homework booklet] and did not understand what was written." (P7).* | "*I had quite a lot of side effects [of the medication] and mindfulness helped me with those. (...) I would just go and do an exercise quietly to get a grip on everything again (…) Because I feel all kinds of things, but where do I feel this now? (...) I usually did the body scan.” (P14).* |

Table S3: Barriers and facilitators

| **General changes** |  |
| --- | --- |
| **Emotion regulation** | *"Worrying? (...) I know exactly how to deal with it now. (…) I didn't have all that before. If I started it, I could go on for hours. But that's just gone now." (P9).* |
| **Thoughts** | *"My primary reaction is to blame myself. But because of the mindfulness I can quicker recognize when I am doing that and I can turn it around." (P14*). |
| **Behavior** | *"So in the beginning I really kept going even if I was actually very tired or had a bad day. (...) And now I can indicate much better that at some point I'm like, hey you know what, it's done, we'll make a new appointment." (P14).* |
| **Interpersonal** | "*I was always someone who couldn't talk about my problems, but now I talk about them very easily since I've been there [MBCT training]." (P9).* |
| **Specific to population** |  |
| **Pain** | *"I get a new complaint, now something with my right hip, and then I can just look at it quietly, like okay (...) We can't do anything about it, okay, that's the case. Stress doesn't help me, I can make a fuss about it, but then the pain only gets worse, (...) then I get more tense, I get more pain." (P14)*. |
| **Addiction** | *"It's just a strong craving to use it (...). Only when you think a little more about the consequences and try to create a picture of what's going to happen after, you actually start linking negative aspects to it. (...) And slowly that gets a bit stronger and the craving decreases." (P2).* |

Table S4: Process of change during MBCT

**References in the appendix**

Bohlmeijer, E., ten Klooster, P. M., Fledderus, M., Veehof, M., & Baer, R. (2011). Psychometric properties of the five facet mindfulness questionnaire in depressed adults and development of a short form. *Assessment, 18*(3), 308-320. doi:10.1177/1073191111408231

de Beurs, E., Van Dyck, R., Marquenie, L. A., Lange, A., & Blonk, R. W. B. (2001). De DASS: een vragenlijst voor het meten van depressie, angst en stress. *Gedragstherapie, 34*(1), 35-54.

Kraaimaat, F. W., & Evers, A. W. (2003). Pain-coping strategies in chronic pain patients: psychometric characteristics of the pain-coping inventory (PCI). *Int J Behav Med, 10*(4), 343-363. doi:10.1207/s15327558ijbm1004_5

Lamers, S. M., Westerhof, G. J., Bohlmeijer, E. T., ten Klooster, P. M., & Keyes, C. L. (2011). Evaluating the psychometric properties of the Mental Health Continuum-Short Form (MHC-SF). *J Clin Psychol, 67*(1), 99-110. doi:10.1002/jclp.20741

Raes, F., Pommier, E., Neff, K. D., & Van Gucht, D. (2011). Construction and factorial validation of a short form of the Self-Compassion Scale. *Clin Psychol Psychother, 18*(3), 250-255. doi:10.1002/cpp.702
